# Filter Paper Blood Spot Enzyme Linked Immunoassay for Adiponectin and Application in the Evaluation of Determinants of Child Insulin Sensitivity

**DOI:** 10.1371/journal.pone.0071315

**Published:** 2013-08-01

**Authors:** Richard M. Martin, Rita Patel, Emily Oken, Jennifer Thompson, Alexander Zinovik, Michael S. Kramer, Konstantin Vilchuck, Natalia Bogdanovich, Natalia Sergeichick, Ying Foo, Nina Gusina

**Affiliations:** 1 School of Social and Community Medicine, University of Bristol, Bristol, United Kingdom; 2 MRC Centre for Causal Analyses in Translational Epidemiology, School of Social and Community Medicine, University of Bristol, Bristol, United Kingdom; 3 University of Bristol/University Hospitals Bristol NHS Foundation Trust, National Institute for Health Research Bristol, Nutrition Biomedical Research Unit, University of Bristol, Bristol, United Kingdom; 4 Department of Population Medicine, Harvard Medical School and Harvard Pilgrim Health Care Institute, Boston, Massachusetts, United States of America; 5 National Research and Applied Medicine, Mother and Child Centre, Minsk, Republic of Belarus; 6 Department of Epidemiology, Biostatistics and Occupational Health, McGill University, Montreal, Quebec, Canada; 7 Department of Pediatrics, McGill University, Montreal, Quebec, Canada; Sapienza, University, Italy

## Abstract

**Background:**

Adiponectin is an adipocyte-derived hormone that acts as a marker of insulin sensitivity. Bloodspot sampling by fingerstick onto filter paper may increase the feasibility of large-scale studies of the determinants of insulin sensitivity. We first describe the validation of an enzyme-linked immunoassay (ELISA) for quantifying adiponectin from dried blood spots and then demonstrate its application in a large trial (PROBIT).

**Methods:**

We quantified adiponectin from 3-mm diameter discs (≈3 µL of blood) punched from dried blood spots obtained from: i) whole blood standards (validation); and ii) PROBIT trial samples (application) in which paediatricians collected blood spots from 13,879 children aged 11.5 years from 31 sites across Belarus. We examined the distribution of bloodspot adiponectin by demographic and anthropometric factors, fasting insulin and glucose.

**Results:**

In the validation study, mean intra-assay coefficients of variation (n = 162) were 15%, 13% and 10% for ‘low’ (6.78 µg/ml), ‘medium’ (18.18 µg/ml) and 'high’ (33.13 µg/ml) internal quality control (IQC) samples, respectively; the respective inter-assay values (n = 40) were 23%, 21% and 14%. The correlation coefficient between 50 paired whole bloodspot versus plasma samples, collected simultaneously, was 0.87 (95% CI: 0.78 to 0.93). Recovery of known quantities of adiponectin (between 4.5 to 36 µg/ml) was 100.3–133%. Bloodspot adiponectin was stable for at least 30 months at −80°C. In PROBIT, we successfully quantified fasting adiponectin from dried blood spots in 13,329 of 13,879 (96%) children. Mean adiponectin (standard deviation) concentrations were 17.34 µg/ml (7.54) in boys and 18.41 µg/ml (7.92) in girls and were inversely associated with body mass index, fat mass, triceps and subscapular skin-fold thickness, waist circumference, height and fasting glucose.

**Conclusions:**

Bloodspot ELISA is suitable for measuring adiponectin in very small volumes of blood collected on filter paper and can be applied to large-scale studies.

## Introduction

There is substantial interest in large-scale epidemiology studies of the genetic and environmental determinants of insulin resistance,[1]–[10] which may inform strategies for the prevention of insulin resistance and its sequelae. [11] Adiponectin is an adipocyte-derived hormone that circulates in high concentrations in humans, moderating glycemia, lipidemia, endothelial dysfunction and proinflammatory mechanisms.[12]–[15] Higher levels of circulating adiponectin are inversely associated with obesity, especially central obesity, as well as hyperlipidemia, insulin resistance, β–cell dysfunction, and intramyocellular lipid accumulation,[16]–[23] both in children and in adults with risk of cardiovascular events.[13], [24]–[26] These relationships, the fact that adiponectin levels are not materially affected by time of day or eating, and the low long-term intra-individual variation, [27] indicate that adiponectin could act as an integrated quantitative measure of insulin sensitivity for use in epidemiology studies. [20], [28].

Important challenges in large-scale epidemiology include non-acceptance of venepuncture especially by children and/or their parents; the costs, safety and logistics of serum or plasma separation by centrifugation; and frozen storage and transport of aliquots. An alternative procedure to venepuncture involves capillary puncture of the finger pulp to collect whole blood spots that are then dried on filter paper. [29], [30] The major advantages of dried blood spot sampling are minimal training, lower cost than venepuncture, acceptability to parents and children, [30] negligible processing requirements (cards must be air dried), low biohazard risk because samples cannot shatter or leak, and the ease of storage and transport of filter paper cards. [29], [31].

As well as large-scale use of dried blood spots in paediatric screening programs for rare inherited disorders, there is widespread interest in developing and validating a wide range of bloodspot assays, [32] including adiponectin, [33] for population research. We first describe the validation of an enzyme-linked immunoassay (ELISA) for quantification of adiponectin from dried blood spots, which offers the important advantage that results can be read on universally available microtitre plate readers, without the need for regulatory approvals to use radioisotopes or more specialised and costly measuring equipment. Development and validation of an ELISA blood spot assay offers a simple, convenient and novel alternative method of measuring adiponectin in large-scale epidemiology studies in a wide range of settings. We then show the successful practical application of our dried blood spot assay in a large-scale, multicentre trial (the Promotion of Breastfeeding Intervention Trial, PROBIT [34]) by describing the distribution of fasting adiponectin levels by demographic and anthropometric factors, fasting insulin and glucose measured in over 13,000 children aged 11.5 years from 31 polyclinics distributed across the Republic of Belarus.

## Materials and Methods

### Validation Study

#### Materials

Blood samples were collected onto Food and Drug Administration approved Whatman 903 filter paper cards, [35] pre-printed with eight 10 mm diameter circles, standardized to absorb blood in a homogeneous manner so that uniform punches from any section of the sample yield the same quantity of blood. [36] We used an existing commercial kit, originally designed for use on serum or plasma (adiponectin human ELISA, EIA4177, DRG International Inc, New Jersey). The kit quantifies adiponectin using a solid phase, two-site enzyme-linked immunoassay, based on the direct sandwich technique in which two monoclonal antibodies are directed against separate antigenic determinants on the adiponectin molecule. Instruments used included an automatic filter paper card puncher (Wallac DBS (Dried Blood Spot) Puncher, product number: 1296-071, Perkin Elmer, USA), a microplate washing device (DELFIA Washer-Diskremove, product number: 1296-0010 Perkin Elmer, USA), a plate shaker (DELFIA Plateshaker, product number:1296-003, Perkin Elmer, USA) and a microplate reader (VICTOR3 Multilabel Reader, product number: 1420-012, Perkin Elmer, USA).

#### Preparation of bloodspot adiponectin standards and internal quality control samples

We prepared whole blood adiponectin standards and internal quality control samples to minimize matrix differences and maximize comparability between the standards, quality control samples and the trial samples. For the standards, we collected whole blood by phlebotomy from a volunteer, centrifuged it, established the adiponectin concentration in plasma, double diluted it in phosphate buffered saline (PBS) and 1% human albumin to obtain levels of 3.75, 7.5, 15.0 and 30.46 µg/ml (top standard). We then mixed each dilution with washed red blood cells (in proportions 60∶40 for a 0.40 haematocrit) and applied drops to Whatman 903 filter paper. Internal quality control samples were based on whole blood taken from 3 volunteers (with low, medium and high values) that was applied to Whatman 903 filter paper and the values assigned from the mean of 24 replicates of the assay.

#### Assay procedure

All frozen blood spot cards (see sample collection below) were brought to room temperature before being removed from the plastic bags for assaying. For each child, one 3-mm disc (≈ 3 µL of blood) was punched from the blood spots (of the standards, quality control samples and the trial samples) using an automatic puncher into 96-well microtitre plates, precoated with a monoclonal anti-human adiponectin antibodies. Adiponectin was extracted from the disc by overnight incubation in a refrigerator with 300 µL of assay buffer (0.05 M Phosphosaline containing 0.025 M EDTA, 0.08% Sodium Azide, 1% BSA). Following incubation, the wells were washed 3 times with wash solution (Tris-buffered saline) to remove unbound enzyme labelled antibody and 20 µL pre-titered biotinylated mouse anti-human adiponectin antibody was added to each well and incubated for 2 hours at room temperature on the plate shaker. Bound conjugate was detected by incubation with 100 µL of enzyme solution (pre-filtered streptavidin-horseradish peroxidise conjugate) and 100 µL of substrate solution (3,3′,5′5′-tetramethylbenzidine in buffer, TMB), before adding 100 µL of stop solution (0.3 M HCl) to terminate the peroxidase/TMB reaction and give a colorimetric endpoint that was read within 5 minutes at 450 and 590 nm. The difference in absorbance units was plotted against the assigned concentration in µg/ml of the whole blood filter paper standard values using a sigmoidal 4- or 5-parameter logistic equation on the log scale on both x and y axes. The concentration of the samples was read from the fitted standard curve. Samples with adiponectin levels above 40 µg/mL but ≤60 µg/ml (3.3%) were reanalyzed by a 1∶2 dilution and those >60 µg/mL (0.7%) reanalyzed by a 1∶4 dilution**.** Adiponectin values between the top standard (30 µg/mL) and 40 µg/ml were assigned the observed value, after determining amongst a sample of 69 children that repeating the assay using double dilution for these levels made no material difference to the observed values. Samples with adiponectin levels less than the lowest standard (3.75 µg/ml, 0.8%) were repeated.

#### Inter- and intra-assay imprecision

Inter- and intra-assay imprecision was assessed using internal quality control samples at three levels: ‘low’ (6.78 µg/ml), ‘medium’ (18.18 µg/ml) and 'high’ (33.13 µg/ml). Intra-assay imprecision was established from duplicate analysis (n = 162) and inter-assay imprecision was determined from 40 separate runs over a period of 29 months. Inter-assay samples were run in duplicate and both intra- and inter-assay imprecision include between-spot extraction variations.

#### Stability

To assess stability at −80°C over the long-term, the whole blood samples used to provide the internal quality controls were spotted onto Whatman 903 cards, cut into strips containing one blood spot per strip, and frozen at −80°C. Initially with every run (for 34 weeks) and then at regular intervals for a total period of 30 months, a different strip was thawed (to ensure levels were based on only one thaw) and the blood spot adiponectin value measured in duplicate.

#### Recovery

We assessed recovery by diluting plasma with an adiponectin concentration of 36 ug/ml with buffered saline to concentrations of 18, 9, 4.5 and 0 ug/ml, mixing the plasma with erythrocytes in the ratio 60∶40, spotting onto Whatman 903 cards and determining the values retrieved after applying the blood spot assay protocol in triplicate.

#### Comparison between plasma and dried blood spots

We compared adiponectin concentrations in plasma (measured using the adiponectin human ELISA, EIA4177, DRG International Inc, New Jersey) with concentrations measured from dried blood spots, collected simultaneously from 50 children in Minsk. Whole blood samples were allowed to stand at room temperature for 2–4 hours then centrifuged at 3000 rpm for 5 minutes before separating the plasma. Blood spots and plasma were stored at −80°C and brought to room temperature before assaying, performed in duplicate for both plasma and dried blood spot samples.

### Application of Methods

#### Distribution of adiponectin by demographic and clinical characteristics

We followed up 13,879 children aged 11.5 years (interquartile range 11.3–11.8 years) who were participants in a multicentre trial of a breastfeeding promotion intervention involving 31 polyclinics (39 paediatricians) located throughout the Republic of Belarus. [34] Children who were eligible for the trial (healthy, term newborns whose mother initiated breastfeeding) were originally recruited at birth between 1996 and 1997, [34] and have been followed up intermittently since then, including the 11.5 year follow-up reported here between 2008–2010. At 11.5 years, dried blood spots were collected from the children as described below. The children also had fasted insulin measured from dried blood spots as described previously, [37] whole blood fasting glucose measured by glucometer (Roche ACCU-CHEK Advantage meter system, F. Hoffmann-La Roche AG, Basel, Switzerland) and the following physical measurements (amongst others not reported here) were made by study paediatricians: Tanner pubertal stage, height, weight, body mass index (BMI, weight (kg)/height (m)^2^), skinfold thicknesses, waist circumference and leg-to-leg bioimpedence (Tanita TBF 300GS, Tanita Europe BV, Netherlands) for calculation of fat-mass. Birthweight had been collected when the children were recruited into the trial at birth in 1996/1997. [34].

#### Sample collection

The protocol for sample collection, quality assurance processes, and the detailed training received by the paediatricians in obtaining blood spots, has been reported in detail elsewhere. [37] In brief, children in the PROBIT trial follow-up were asked to fast for at least 8 hours. Up to 8 drops of fingertip capillary blood were obtained by trained paediatricians using a sterile, single-use disposable lancet device (Roche ACCU-CHEK Safe-T-Pro Plus lancet, Roche Diagnostics Corp., Indianapolis, USA) and applied to the pre-printed circles to form one discrete spot per drop, following specific guidelines. [35], [38] The blood spots were air-dried and then placed in low gas-permeable zip-closure plastic bags with desiccant packages. [36] These were stored initially in –20°C freezers at the 31 polyclinics, prior to transfer in cool boxes packed with freezer packs to −80°C freezers at the central laboratory in Minsk. The samples were stored at −20°C for a median of 1.6 months (IQR 1.0–5.0 months) and at −80°C for a median of 16.9 (IQR 11.9–20.2) months. At the central laboratory in Minsk, 432 samples were assayed in 5 plates per week, using 4 separate assay lots. Assays were run in singleton and repeated if they failed quality control parameters. It took 34 weeks to complete the study.

#### Audit

Once data collection was complete, we conducted audit visits to assess the inter-observer reproducibility, an important step given that the pediatricians were not blinded to randomization status. For each of the 39 pediatricians, 1–5 children were randomly selected for the audit, for a total of 127 audited children with baseline and repeat adiponectin values. So that all children seen in follow-up were eligible for selection, the audit was carried out after completion of primary data collection, an average of 1.3 years (range 0.2 to 2.4 years) after the initial clinic visit. The audit was carried out by one of five Minsk-based pediatricians not involved in primary data collection and blinded to the measures obtained at the initial visit but not to experimental or control status. Because of the time elapsed between the audit and initial visits, results were compared using Spearman correlation coefficients.

#### Ethics

The study received ethical approval from McGill University Health Centre Research Ethics Board; the Human Subjects Committee at Harvard Pilgrim Health Care; and the Avon Longitudinal Study of Parents and Children Law and Ethics Committee. The conduct of the study was also approved by the Ministry of Health of the Republic of Belarus. A parent or legal guardian provided written informed consent and all children provided written assent. The trial registration number is ISRCTN37687716 for Current Controlled Trials and NCT01561612 for Clinicaltrials.gov.

#### Statistical analysis

Calibration curves were constructed with Multicalc software (Wallac, Turku, Finland), using the log scale for both x and y axes. To assess the relationship between adiponectin values from plasma versus whole blood spot samples collected simultaneously, we computed the correlation coefficient (95% confidence interval) and constructed a Bland-Altman plot of the difference between the plasma and bloodspot adiponectin values (y-axis) against the mean of these two values (x-axis). Using data from the PROBIT study, we performed linear regression accounting for clustering by polyclinic to investigate relationships of adiponectin levels with child age, location of polyclinic (urban/rural; East/West Belarus), puberty as measured by Tanner stage, measures of general (BMI, fat mass, peripheral (triceps skinfold thickness) and central (subscapular skinfold thickness, waist circumference) adiposity, height, birthweight and fasting glucose, and insulin. We calculated sex-specific associations and performed a likelihood ratio test for interactions between sex and the demographic, glucose, insulin and anthropometric factors on adiponectin. We also performed a likelihood ratio interaction test of whether the association of adiponectin with insulin levels varied by whether the children were overweight/obese versus normal weight. Analyses were undertaken using Stata version 11 (StataCorp LP, Texas) and SAS version 9.3 (SAS Institute, Inc., Cary, North Carolina).

## Results

**Figure 1:**
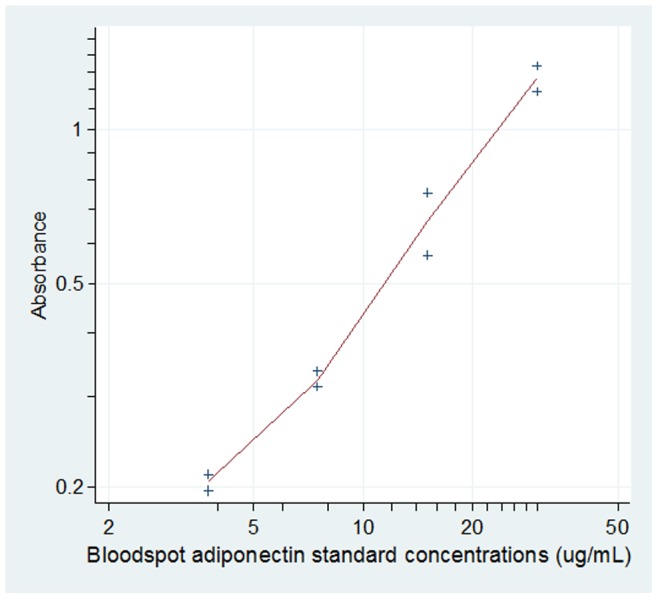
Typical calibration curve. Whole bloodspot adiponectin standards at 3.75, 7.5, 15.0, 30.46 µg/ml.

A typical calibration curve is shown in **Figure 1** **.** The precision of the dried blood spot extraction and consecutive determination by ELISA is summarised in **Table 1**. Mean intra-assay (n = 162) coefficients of variation were 15%, 13% and 10% for ‘low’ (6.78 µg/ml), ‘medium’ (18.18 µg/ml) and 'high’ (33.13 µg/ml) IQC samples, respectively; the respective inter-assay values (n = 40) were 23%, 21% and 14%.

**Table 1:** Intra- and inter-assay imprecision of dried whole bloodspot adiponectin assays.

Sample	Mean adiponectin (µg/ml)	Mean standard deviation of repeated measures	Coefficient of variation
*Intra-assay (n = *162*)*			
Low	6.78	1.00	15%
Medium	18.18	2.41	13%
High	33.13	3.47	10%
*Inter-assay (n = 40)*			
Low	6.78	1.54	23%
Medium	18.18	3.73	21%
High	33.13	4.53	14%

**Figure 2:**
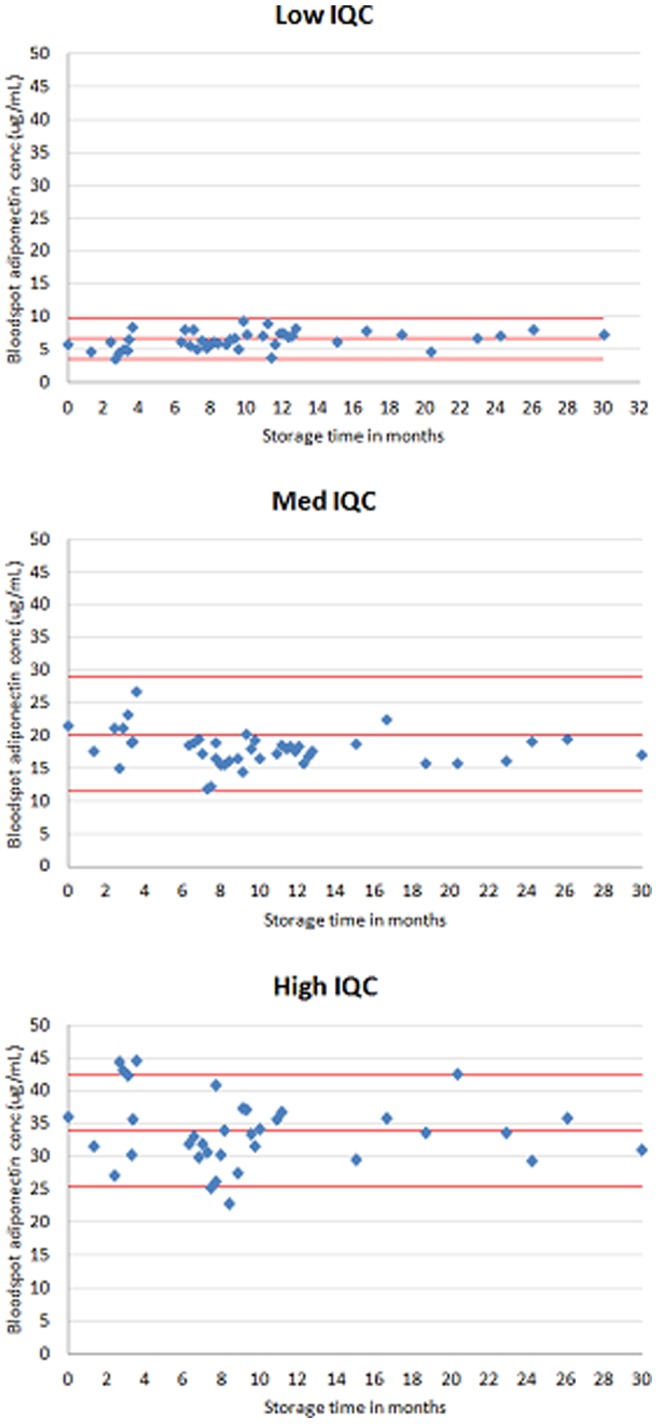
Stability of adiponectin concentrations (at A. low, B. medium and C. high values) measured from dried blood spots and stored at −80°C for 30 months. Adiponectin concentrations of internal quality control (IQC) bloodspots (low, medium and high values) analysed at regular intervals over 30 months. For the ‘low’ IQC sample (Fig. 2A), the middle red line is the mean adiponectin concentration and the upper and lower red lines are the 95% reference range (calculated from the standard deviation of 24 replicates); the respective lines showing the mean and 95% reference range for the ‘medium’ (Fig. 2B) and ‘high’ (Fig. 2C) IQC samples are also shown.

The results of the 30 month long-term stability study at −80°C are shown in **Figure 2**. Over the 30 months, most values were within the 95% reference range established from 24 replicates of the time 0 value, although there were month-by-month fluctuations in line with the inter-assay imprecision of measurements. In linear regression analyses, adjusted for lot number, there was no evidence of any association between storage time at −80°C and levels of adiponectin for the low (change in adiponectin levels per month = 0.04 µg/ml; 95% CI: −0.04 to 0.12; p = 0.33), medium (0.05 µg/ml; 95% CI: −0.12 to 0.22; p = 0.53), or high (0.07 µg/ml; 95% CI: −0.30 to 0.44; p = 0.70) internal quality control samples.

### Comparison of Samples and Recovery

**Figure 3:**
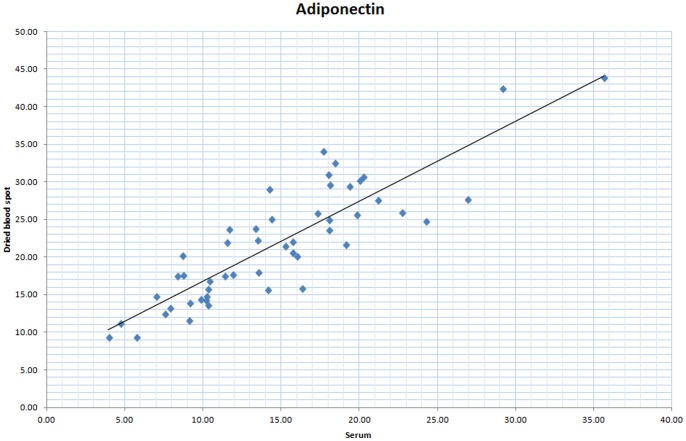
Comparison of adiponectin concentrations measured from 50 paired plasma and whole bloodspot samples.

**Figure 4:**
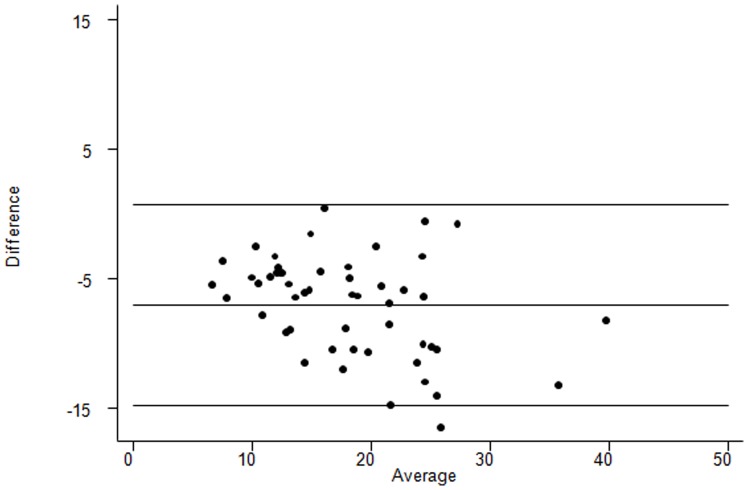
Bland-Altman plot for plasma adiponectin minus bloodspot adiponectin values in 50 paired samples. Limits of agreement (reference range for the difference): −14.8 to 0.68 µg/ml. Mean difference in adiponectin between plasma minus blood spot samples: −7.1 µg/ml (95% CI: −8.2 to −6.0). Range of average values: 6.7 to 39.8 µg/ml.

Comparison of 50 paired fasted whole bloodspots and plasma samples samples collected simultaneously gave a correlation coefficient of 0.87 (95% CI: 0.78 to 0.93) (**Figure 3** **)**, the range of plasma values being 3.9 to 35.7 µg/ml. The Bland Altman plot shows that the 95% limits of agreement were between −14.8 to 0.68 µg/ml with a mean difference (plasma minus blood spot) across the range of values of −7.1 µg/ml (CI −8.2 to −6.0), indicating that blood spot measurement of adiponectin was higher on average than plasma measurement (**Figure 4**). Inference was similar when the Bland-Altman plot was based on the ratio of bloodspot to plasma adiponectin values **(Figure S1)** Recovery of known quantities of adiponectin (between 4.5 to 36 µg/ml) was 100.3–133% (**Table 2**).

**Table 2:** Recovery of adiponectin from five different dried blood spot concentrations.

Adiponectin in whole blood (µg/ml)	Adiponectin in dried blood (µg/ml)	Recovery, %
0	0.34	–
4.5	5.84	130%
9.0	9.025	100.3%
18	20.14	111.9%
36	48.04	133%

### Associations of Adiponectin with Demographic and Clinical Variables

**Figure 5:**
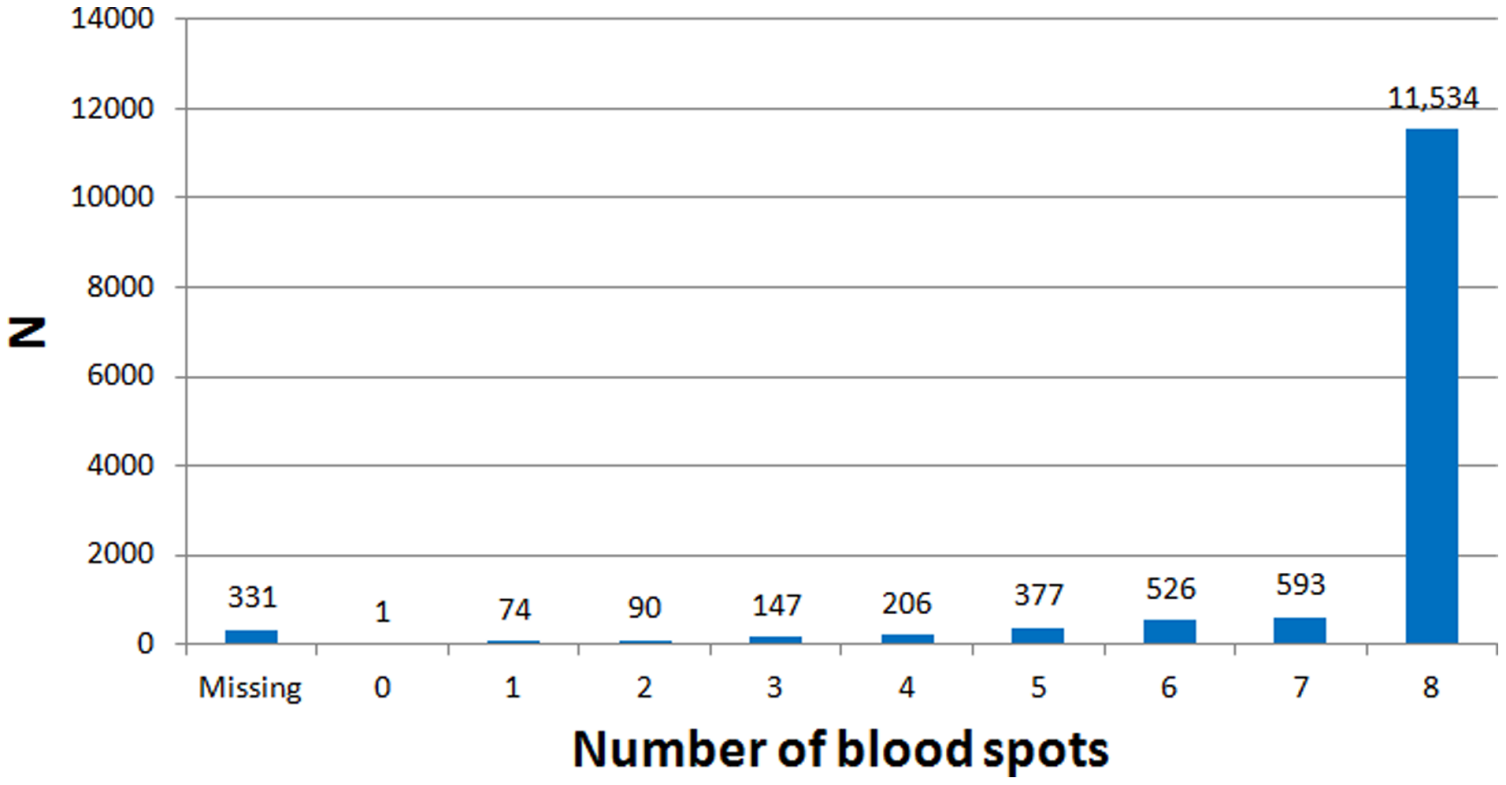
Number of blood spots per child collected in the PROBIT study when the children were aged 11.5 years.

Out of a total of 13,879 children who were approached, 13,547 (97.6%) provided at least one acceptable dried blood spot sample (**Figure 5**). Amongst the 13,547 children, the paediatricians collected a median of 8 blood spots, with 85.1% (11,534) of children assessed by the central laboratory in Minsk as having had 8 acceptable quality blood spots collected. We assayed a total of 13,329 children who fasted for 8 or more hours for adiponectin.

**Table 3** shows adiponectin levels in boys and girls by demographic and clinical variables. Mean adiponectin (standard deviation) concentrations were 17.34 µg/ml (7.54) in boys and 18.41 µg/ml (7.92) in girls (p for gender difference<0.001). Concentrations of adiponectin were inversely associated with Tanner stage by public hair assessment (boys). Adiponectin concentrations were also inversely associated with body mass index, fat mass, mean triceps and subscapular thicknesses, waist circumference, height and fasting glucose concentrations. There was evidence of an interaction between sex and body mass index, mean triceps thickness and mean subscapular thickness (p for interaction<0.01), although the direction of the effects in both boys and girls was inverse. There was little evidence that the null association of adiponectin with insulin levels varied by whether the children were overweight/obese versus normal weight (p for interaction = 0.15). The audit results between initial clinic results and blinded repeat (audit) measures of adiponectin showed a Spearman correlation coefficient of 0.33.

**Table 3:** Adiponectin levels in boys and girls by demographic and clinical characteristics, N = 13,329.

	Adiponectin levels (µg/ml) and age-adjusted difference in adiponectin(95% CI) per unit of demographic/clinical variable				
	N	Girls	N	Boys	p for sexinteraction
**Age group (yrs)**					
10 – <11	407	17.6 (7.3)	422	17.0 (7.5)	
11 – <12	4949	18.6 (7.9)	5191	17.5 (7.6)	
12 – <13	1012	18.2 (7.9)	1108	17.0 (7.3)	
13 – <14	110	14.0 (7.3)	130	13.8 (5.1)	
Change in adiponectin per year^a^		−0.50 (−1.84, 0.83)		−0.61 (−1.35, 0.13)	0.77
**Location**					
Rural	2738	18.6 (7.9)	2860	17.5 (7.8)	
Urban	3740	18.3 (7.9)	3991	17.2 (7.3)	
*Difference in adiponectin* a		0.34 (−1.00, 1.68)		0.33 (−0.78, 1.45)	0.96
East	3114	18.3 (7.9)	3179	17.3 (7.5)	
West	3364	18.5 (7.9)	3672	17.3 (7.5)	
*Difference in adiponectin* a		−0.17 (−1.48, 1.14)		−0.02 (−1.16, 1.11)	0.60
**Tanner stage by breast (girls)/genitalia (boys)**					
I	745	18.4 (7.5)	897	16.9 (7.3)	
II	3323	18.7 (8.1)	3699	17.6 (7.7)	
III	2080	18.1 (7.8)	1995	17.0 (7.3)	
IV/V	325	17.4 (6.9)	252	17.9 (7.9)	
*Change in adiponectin per category* a		−0.37 (−0.87, 0.14)		−0.001 (−0.61, 0.61)	0.20
**Tanner stage by pubic hair assessment**					
I	1819	18.6 (8.0)	3440	17.6 (7.7)	
II	3035	18.5 (8.0)	2808	17.3 (7.4)	
III	1434	17.9 (7.6)	546	16.4 (6.8)	
IV/V	185	18.7 (7.0)	49	15.0 (6.4)	
*Change in adiponectin per category* a		−0.24 (−0.71, 0.23)		−0.50 (−0.90, −0.10)	0.15
**Body mass index (kg/m^2^)** b					
Normal weight	5627	18.70 (7.93)	5668	17.61 (7.57)	
Overweight	607	17.21 (7.68)	738	16.49 (7.38)	
Obese	240	14.97 (6.85)	439	15.53 (7.02)	
*Change in adiponectin per category* a		−1.73 (−2.06, −1.39)		−1.06 (−1.42, −0.70)	0.003
**Fat mass (kg)**					
<90^th^ centile	5913	18.6 (7.9)	6128	17.5 (7.5)	
≥90^th^ centile^d^	652	16.4 (7.5)	688	15.9 (7.3)	
*Difference in adiponectin* a		−2.19 (−2.65, −1.72)		−1.60 (−2.29, −0.90)	0.10
**Triceps thickness (mm)**					
<90^th^ centile	5900	18.6 (8.0)	6149	17.5 (7.5)	
≥90^th^ centile^d^	724	16.5 (7.0)	699	16.4 (7.3)	
*Difference in adiponectin* a		−2.15 (−2.93, −1.38)		−1.03 (−1.71, −0.35)	0.004
**Subscapular thickness (mm)**					
<90^th^ centile	6214	18.5 (7.9)	6567	17.4 (7.5)	
≥90^th^ centile^d^	258	15.1 (6.4)	282	15.5 (7.0)	
*Difference in adiponectin* a		−3.40 (−4.14, −2.67)		−1.91 (−2.64, −1.19)	0.001
**Waist circumference (cm)**					
<90^th^ centile	6106	18.6 (7.9)	6704	17.5 (7.5)	
≥90^th^ centile^c^	369	15.6 (7.0)	305	15.3 (7.2)	
*Difference in adiponectin* a		−2.23 (−2.67, −1.79)		−1.53 (−2.21, −0.85)	0.08
**Height (cm)** e					
Q1	2036	18.9 (8.1)	2013	17.7 (7.5)	
Q2	1627	18.3 (8.0)	1822	17.7 (7.6)	
Q3	1460	18.2 (7.9)	2022	17.1 (7.5)	
Q4	1355	17.9 (7.5)	992	16.5 (7.4)	
*Change in adiponectin per 10 cm* a		−0.47 (−0.86, −0.07)		−0.49 (−0.85, −0.14)	0.79
**Birthweight (g)** f					
Q1	1819	18.0 (7.9)	1748	17.0 (7.4)	
Q2	1466	18.4 (7.8)	1851	17.4 (7.5)	
Q3	1579	18.6 (8.0)	1776	17.5 (7.6)	
Q4	1614	18.7 (7.9)	1476	17.4 (7.6)	
*Change in adiponectin per 100g* a		0.07 (0.03, 0.11)		0.03 (−0.02, 0.08)	0.16
**Glucose(mmol/l)**					
Q1	1569	19.5 (8.0)	1979	17.9 (7.5)	
Q2	1601	19.1 (8.1)	1337	17.9 (7.9)	
Q3	1521	17.9 (7.8)	1628	17.3 (7.5)	
Q4	1778	17.3 (7.6)	1902	16.5 (7.3)	
*Change in adiponectin per SD increase in glucose* a		−0.55 (−0.89, −0.22)		−0.55 (−0.89, −0.21)	0.96
**Insulin (mU/L)**					
Q1	1570	17.6 (7.6)	1626	16.8 (7.2)	
Q2	1578	18.6 (7.8)	1630	17.4 (7.4)	
Q3	1578	19.0 (8.3)	1628	17.7 (7.6)	
Q4	1582	18.4 (7.8)	1638	17.7 (7.9)	
*Change in adiponectin per SD increase in insulin* a		0.20 (−0.03, 0.44)		0.004 (−0.19, 0.20)	0.07

SD = standard deviation; CI = confidence interval.

a Linear regression coefficient and 95% CI (age-adjusted, accounting for clustering by polyclinic).

b Normal weight, overweight and obese as defined by the International Obesity Task Force BMI cut-offs. [44].

c Centiles derived from NHANES European age and sex-specific thresholds (reference) [45].

d Centile derived from sample.

e Quartiles derived from NHSR age-and sex-specific thresholds [46].

f Quartiles derived from sample.

## Discussion

We have shown that adiponectin in dried blood spots is stable and can be reliably quantified on a very large-scale with modest infrastructure, making the method useful for population-based studies of adiposity, insulin resistance, diabetes and cardiovascular risk in a variety of settings. Our data support a previous paper reporting on the quantification of adiponectin in dried blood spots from neonates in a small retrospective case-control study (n = 124). [33] The advantages of fingerstick blood collection onto filter paper include ease of collection, processing, storage and transport, thus providing a valuable research tool for large epidemiology studies, particularly those that are geographically dispersed.

The dried blood spot methods showed acceptable analytical performance characteristics and good agreement with the conventional plasma adiponectin assay across the distribution of adiponectin values. There are several sources of variation unique to bloodspot sampling and the assay that could explain differences between bloodspot versus plasma. For example, proper placement of the whole blood sample on the filter paper is critical and errors can be introduced if blood is blotted or smeared rather than drawn onto the filter paper by capillary action. [32] We minimised this source of error by rigorous training and regular feedback. [37] Pre-printed circles on the filter paper were used to help guide the paediatricians to position the bloodspot. To maximise stability, samples were placed in zip-closure bags packed with dessicant and frozen at −20°C promptly after drying to reduce the chances of degradation. These sources of variation are not seen with venepuncture sampling and the coefficients of variation reported in **Table 1** were larger than those reported in the adiponectin kit insert (adiponectin human ELISA, EIA4177, DRG International Inc, New Jersey): 7.4%, 0.9% and 1.8% for ‘low’ (17.73 µg/ml), ‘medium’ (29.13 µg/ml) and 'high’ (39.10 µg/ml) serum samples, respectively; the respective inter-assay values were 8.4%, 2.4% and 6.2%. The haematocrit level was set to 0.4 in the standards but not adjusted for in each sample; any sample differences would introduce errors. The variation seen in **Figure 2** is broadly in line with the imprecision suggested in **Table 1**. Despite unique sources of error, we achieved a good correlation between dried blood spot versus plasma methods of measurement.

In our fieldwork study, we showed that taking capillary blood samples from children is feasible on a large scale, since only 2.4% (n = 332) of 13,879 children approached did not provide a useable sample and the paediatricians were able to take a median of 8 acceptable blood spots amongst those who were sampled. Although we documented slightly greater imprecision than would be expected in plasma or serum samples, our analysis of established associations of adiponectin levels with gender, adiposity measures, glucose and height, were in line with previously published results.[39]–[42] We did not observe an association of adiponectin with fasting insulin levels in our population of 11.5 year olds, in line with others indicating a complex relationship at this age. [41], [42] The mean values in girls and boys that we observed were slightly higher, but in line with other reports (given age, setting, population and sampling variation) **(** **Table 4** **)**.[39]–[42].

**Table 4:** Comparison of adiponectin levels measured in PROBIT with other studies in children of a similar age.

			Adiponectin levels (µg/ml)	
Study (ordered by subjects’ age)	Mean age, years (SD, unless otherwise stated)	Number	Mean/median	SD (unless otherwise stated)
**Girls**				
Ong [42]	8.2 (8.1–8.2)^a^	390	14.0	11.4–17.6^b^
PROBIT	11.5 (11.3–11.8)^a^	6478	18.4	7.9
Okada [39]	11.6 (1.5)	139	15.4	7.1
Punthakee [41]	13	235	10.9	10.4–11.4^c^
Singhal [40]	14.9 (0.9)	160	7.6	5.6–10.6^b^
**Boys**				
Ong [42]	8.2 (8.1–8.2)^a^	449	13.0	11.1–16.0^b^
PROBIT	11.5 (11.3–11.8)^a^	6851	17.35	7.5
Okada [39]	11.6 (1.5)	144	13.7	6.1
Punthakee [41]	13	269	9.1	8.6–9.5^c^
Singhal [40]	15.0 (0.9)	134	6.4	4.7–9.5^b^

SD = standard deviation; m = months;

a median (interquartile range);

b interquartile range;

c 95% CI.

The advantages of filter paper capillary blood sampling, combined with the acceptable analytical performance characteristics of the dried blood spot assays, makes the approach ideal for large-scale epidemiologic applications, including studies of children, whenever the acceptability, stability, cost and safety of liquid sample collection and transportation to distant laboratories may be limiting factors (which may not be the case, however, for small and medium sized studies that are geographically contained). There are caveats, as we have previously pointed out, [37] including the need for adequate training for those taking the bloodspot samples onto filter paper, to avoid several sources of error, and the fact that we have developed the dried bloodspot assay for research and not clinical use, which would require additional investigation. [43].

## Supporting Information

Figure S1 **Bland-Altman plot for the ratio of bloodspot to plasma adiponectin values in 50 paired samples.** Mean ratio of bloodspot to plasma adiponectin values: 1.57 (95% CI: 1.47 to 1.67). Range of ratio values: 0.97 to 2.39.
(PPTX)
