# Supplementary figures and images for: Filter Paper Blood Spot Enzyme Linked Immunoassay for Adiponectin and Application in the Evaluation of Determinants of Child Insulin Sensitivity
Source: PLoS One. 2013 Aug 1;8(8):e71315. doi: 10.1371/journal.pone.0071315 (PMC3731301; doi:10.1371/journal.pone.0071315)

## Slide 1
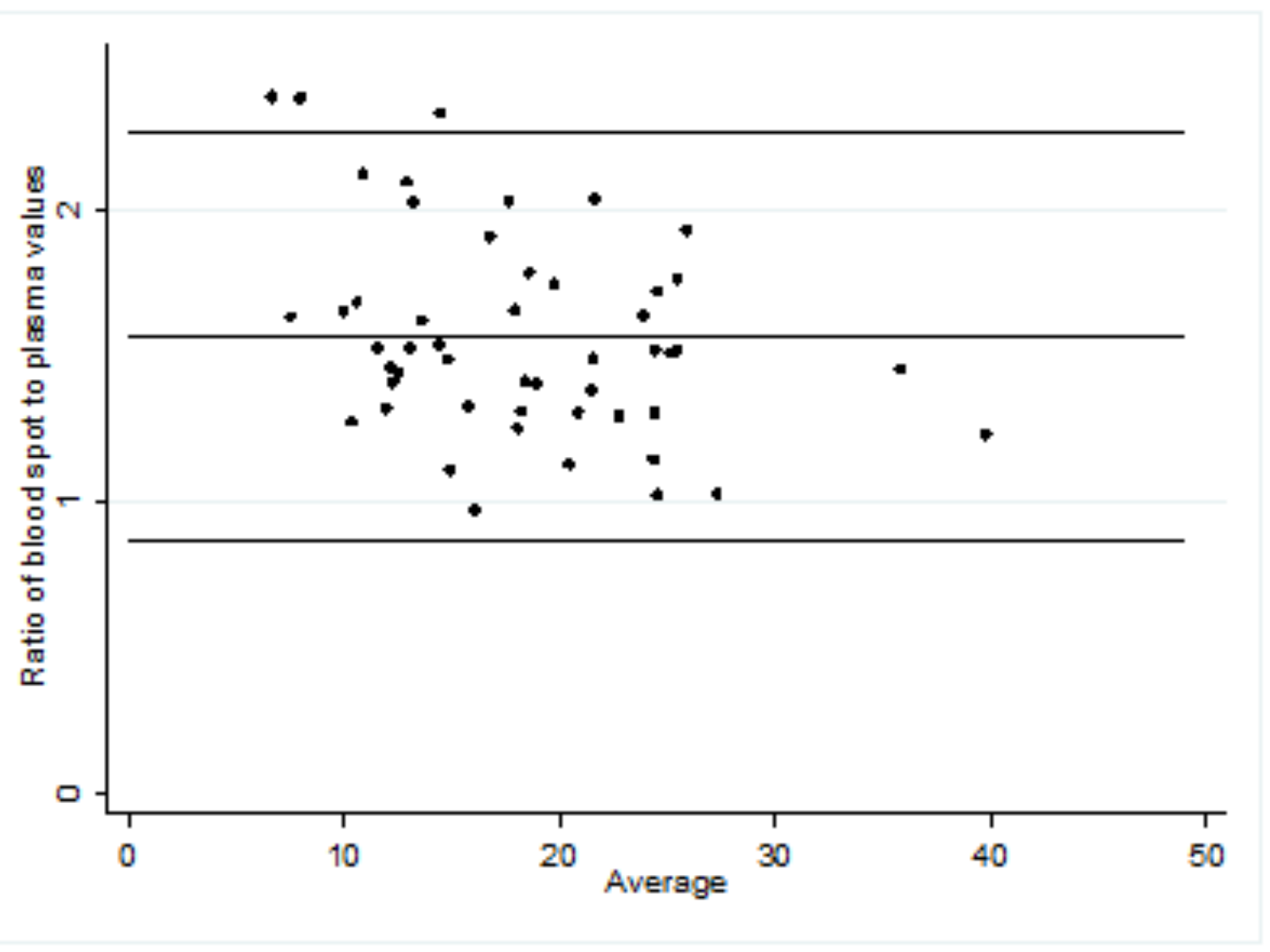

#

Supplement: Figure S1 — Bland-Altman plot for the ratio of bloodspot to plasma adiponectin values in 50 paired samples. Mean ratio of bloodspot to plasma adiponectin values: 1.57 (95% CI: 1.47 to 1.67). Range of ratio values: 0.97 to 2.39. (PPTX) [file pone.0071315.s001.pptx]
